# Platelet-to-lymphocyte ratio might predict the response to dupilumab treatment for patients with nasal polyposis

**DOI:** 10.1186/s40463-023-00660-7

**Published:** 2023-11-25

**Authors:** Faris F. Brkic, David T. Liu, Iris Rücklinger, Nicholas James Campion, Tina Josefin Bartosik, Erich Vyskocil, Victoria Stanek, Aldine Tu, Katharina Gangl, Sven Schneider

**Affiliations:** Department of Otorhinolaryngology and Head and Neck Surgery, Medical University of Vienna, Währinger Gürtel 18-20, 1090 Vienna, Austria

**Keywords:** Dupilumab, Nasal polyps, Chronic rhinosinusitis with nasal polyps, Biomarker

## Abstract

**Background:**

Dupilumab is a monoclonal antibody against interleukin 4 receptor alpha and has proven to be clinically effective in treating patients with chronic rhinosinusitis with nasal polyps (CRSwNP). However, a certain number of patients are non- or partial responders. This study aims to investigate the relevance of inflammatory markers with regard to therapy response to dupilumab in CRSwNP patients.

**Methods:**

All patients with CRSwNP treated with dupilumab at a tertiary healthcare center with available pretreatment inflammatory markers were included. The values of pretreatment neutrophil-to-lymphocyte ratio (NLR) and platelet-to-lymphocyte ratio (PLR) were associated with the outcome. Patients were stratified according to the respective median value (> median was considered high). The binary logistic regression was performed with regard to total treatment response (post-treatment total nasal polyp score (NPS) 0).

**Results:**

A total of 65 CRSwNP patients with available pretreatment peripheral blood values were included in the study. The mean pre- and post-treatment total NPS values were 4.3 ± 1.9 and 1.2 ± 1.6, respectively. High PLR (> 131.2) was independently associated with a 3.9-fold higher probability of reaching the NPS value of 0 in the multivariable analysis. On the other hand, High NLR (> 1.9) did not significantly associate with the outcome.

**Conclusions:**

The current study provides insights into the potential positive predictive value of the high PLR (> 131.2) in CRSwNP patients regarding treatment with dupilumab. There is a need for further prospective studies for validation of these results, especially in cohorts of patients with severe CRSwNP.

## Introduction

Chronic rhinosinusitis with nasal polyps (CRSwNP) affects 1.95–4.4% of the population and represents a significant burden for patients and healthcare system [1–4]. Indeed, the quality of life is affected negatively to a great extent [5]. Notably, for the United States of America (USA) alone, the annual direct and indirect costs of treating CRSwNP were reported to be up to 6 billion United States dollars [6].

Standard treatment options involve topical and systemic corticosteroid therapy as well as endoscopic sinus surgery (ESS). However, recurrent disease after ESS is still a major challenge and long-term corticosteroid treatment is often warranted [7]. Therefore, efforts have been made to identify predictive biomarkers. One study group provided evidence that high eosinophil counts are associated with a better response to corticosteroid treatment [8]. The prognostic value of inflammatory biomarkers, including neutrophil-to-lymphocyte ratio (NLR), platelet-to-lymphocyte ratio (PLR) and monocyte-to-lymphocyte ratio (MLR) has been investigated by several authors [9, 10]. In particular, Boztepe et al. [10] noted that the recurrent CRSwNP after ESS was associated with higher preoperative NLR.

Advances and discoveries of type 2 inflammation profiles driving the CRSwNP pathophysiology facilitated the development of novel treatment options. In particular, dupilumab is a human monoclonal antibody targeting interleukin (IL)-4 and IL-13 signaling. Indeed, exceptional results after Dupilumab treatment have been reported in terms of both objective and subjective outcomes [11]. Despite very good results, reported non-responder rates range up to 40% [12]. Little evidence exists on prognostic markers for dupilumab response in CRSwNP. In particular, Bachert et al. [13] summed up the findings of two phase 3 trials investigating inflammatory biomarkers for CRSwNP patients treated with dupilumab. In particular, there was only a weak or moderate correlation of nasal polyp score (NPS) improvement and absolute eosinophil count, IgE, periostin, and other hematologic markers reflecting type 2 inflammation.

Therefore, easily obtainable predictive markers for Dupilumab response in CRSwNP are warranted. Although the prognostic capacity of inflammatory biomarkers NLR and PLR was already analyzed in patients with chronic rhinosinusitis, no studies assessed the prognostic values of these biomarkers for patients with CRSwNP treated with dupilumab. Therefore, in the current study, we aimed to assess if pretreatment values of NLR and PLR are associated with treatment response to dupilumab in CRSwNP.

## Materials and methods

This retrospective analysis was performed at the Department of Otorhinolaryngology, Head and Neck Surgery, Medical University of Vienna. All CRSwNP patients treated with dupilumab (biweekly 300 mg) between January 1st 2020 and August 31st 2021 were included in the study. Patients were followed up after 1, 2, and 6 months in the outpatient clinic (some deviations occurred due to the coronavirus disease (COVID19) pandemic). Patients with a previous treatment with systemic and current treatment with topical corticosteroids were eligible for dupilumab therapy. Pathologies other than primary CRSwNP were excluded from the analysis. The routine clinical examination included the documentation of the total nasal polyp score (NPS) (also known as Meltzer endoscopic nasal polyp score) (0–8), where the individual (left and right) scores (0–4) were added up. This score was characterized as proposed by Meltzer et al. [14] and was assessed prior to treatment (day 0) and routinely in clinical check-ups during dupilumab therapy. Furthermore, subjective outcome measures were assessed, and included the Sino-nasal Outcome Test (SNOT-22), visual analogue scale (VAS) for sino-nasal symptoms (sense of smell and nasal breathing), and Sniffin’ sticks.

The following variables were extracted from the patient’s medical histories: age, sex, pretreatment NPS (0–8), post-treatment NPS (at least eight weeks after the start of dupilumab treatment), pretreatment absolute neutrophil, platelet, lymphocyte and eosinophil counts in Giga (G) / liter (L) (0–14 days prior to treatment start). Moreover, presence of comorbidities (aspirin-exacerbated respiratory disease (AERD) and asthma) as well as the number of previous ESS were retrieved as well. The values of NLR and PLR were calculated by dividing the absolute counts of corresponding cells. Patients that chose to be followed up elsewhere or without available pretreatment peripheral blood counts were excluded from the study.

### Statistics

The statistical analysis was conducted using the Statistical Package for the Social Sciences (SPSS, IBM Corp. Released 2016. IBM SPSS Statistics for Windows, Version 24.0. IBM Corp., Armonk, NY, USA). Due to the normal data distribution observed in the histograms, we presented descriptive data with mean ± standard deviation (SD). Furthermore, the normal data distribution was confirmed in the Shapiro–Wilk-Test. As the literature was sparse with regard to the prognostic capacity of NLR and PLR in CRSwNP, we stratified the patients into low and high groups, with the median used as the cut-off value (> median was considered as high), which was calculated for both, as well. The improvement in the NPS score was the outcome parameter and was dichotomized into complete NPS reduction (post-treatment NPS 0) and incomplete NPS reduction (post-treatment NPS > 0). In order to assess the association of the inflammatory parameters and the NPS improvement, the chi-square test was utilized. Thereafter, we utilized univariable and multivariable binary logistic regression in order to independently test the markers for their prognostic capacity. Variables with significant results in the univariable analysis were selected and analyzed with confounders (number of prior ESS, asthma, AERD, age, sex, absolute platelet, neutrophil, and eosinophil count) in the multivariable analysis via the entry method. In order to test the prognostic relevance of PLR with regards to improvements of the questionaries, a chi-square test was performed. Improvements in SNOT-22, VAS nasal breathing and sense of smell, and Sniffin’ sticks were stratified into high and low according to the median value. All results at p < 0.05, two-sided, were considered statistically significant.

## Results

### Patients

We included a total of 65 patients with CRSwNP treated with dupilumab in the study period with available pretreatment blood samples and clinical follow-up in our center. Forty-two patients (64.6%) were male, and 23 patients (35.4%) were female. The mean age of the cohort was 51.3 ± 12.7 years. Due to the COVID19 pandemic, there were some deviations from the planned clinical follow-up assessments (1, 2, and 6 months). Therefore, to assess the improvement in NPS, the value after at least two months was considered post-treatment. Importantly, none of the patients were on systemic corticosteroid therapy immediately prior to or during dupilumab treatment. Notably, the vast majority of patients were Caucasian (95.4%, n = 62/65). Table 1 shows detailed patient characteristics including confounders/comorbidities (asthma, AERD). The groups (low NLR vs. high NLR and low PLR vs. high PLR) did not show statistically significant differences in the distribution of Asthma, AERD, and number of prior ESS.

**Table 1:** Patient characteristics

Patients		NLR ≤ 1.9	NLR > 1.9	PLR ≤ 131.2	PLR > 131.2
	n = 65	n = 33	n = 32	n = 33	n = 32
Male/female					
n	42/23	23/10	19/13	26/7	16/16
%	64.6/35.4	69.7/30.3	59.4/40.6	78.8/21.2	50.0/50.0
Age, mean years (SD)	51.3 (12.7)	48.1 (11.0)	54.6 (13.4)	51.6 (12.7)	51.0 (12.6)
Asthma n/%	41/63.1	20/62.5	21/70.0	18/58.1	23/74.2
AERD n/%	32/49.2	19/57.6	13/41.9	15/46.9	17/53.1
Prior ESS, mean number (SD)	2.0 (1.5)	2.0 (1.6)	2.1 (1.4)	2.0 (1.6)	2.1 (1.4)
Pretreatment NPS					
Median	4	4	4	1	4
Min.–max	1–8	1–8	1–8	0–6	1–8

*n* number of patients, *%* percentage of patients, *AERD* aspirin-exacerbated respiratory disease, *ESS* endoscopic sinus surgery, *SD* standard deviation, *NLR* neutrophil-to-lymphocyte ratio, *PLR* platelet-to-lymphocyte ratio, *min.* minimum value, *max.* maximum value

### Nasal polyp score

The mean pretreatment NPS of the whole cohort was 4.3 ± 1.9 (median NPS 4, range 1–8), indicating a mild disease in a majority of patients. Meanwhile, mean NPS value at least eight weeks after Dupilumab treatment for the entire cohort was 1.2 ± 1.6 (median NPS 1, range 0–6) and the mean improvement for the whole cohort of 3.1 ± 1.7 was observed. Total improvement (post-treatment NPS 0) was observed in 32 patients (49.2%). The NPS score did not improve in two patients (3.1%). Table 2 shows patients characteristics stratified for total NPS improvement.

**Table 2:** Characteristics for the cohort stratified by the NPS improvement status

Patients		ntNPSi	tNPSi
	n = 65	n = 33	n = 32
Male/female			
n	42/23	20/13	22/10
%	64.6/35.4	60.6/39.4	68.8/31.3
Age, mean years (SD)	51.3 (12.7)	50.3 (13.5)	52.4 (11.7)
Asthma n/%	41/63.1	17/51.5	24/75.0
AERD n/%	32/49.2	13/39.4	19/59.4
Prior ESS, mean number (SD)	2.0 (1.5)	1.9 (1.5)	2.2 (1.5)
Pretreatment NPS			
Median	4	2	4
Min.–max	1–8	1–6	1–8

*NPS* nasal polyp score, *ntNPSi* no total NPS improvement, *tNPSi* total NPS improvement, *n* number of patients, *%* percentage of patients, *AERD* aspirin-exacerbated respiratory disease, *ESS* endoscopic sinus surgery, *SD* standard deviation, *NLR* neutrophil-to-lymphocyte ratio, *PLR* platelet-to-lymphocyte ratio, *min.* minimum value, *max.* maximum value

### Subjective outcomes

Average improvements in the VAS for sense of smell and nasal breathing (available in 58 and 57 patients, respectively) were 3.5 ± 3.5 and 2.7 ± 3.6 points while the mean improvement in SNOT-22 (available in 41 patients) was 27 ± 19. Lastly, Sniffin’ sticks were performed in 47 patients and a mean improvement of 3.8 ± 3.6 points was calculated.

### Pretreatment blood values

The mean absolute neutrophil and lymphocyte counts were 4.1 ± 1.4 G/L and 2.0 ± 0.6 G/L, respectively. Furthermore, the mean platelet count was 265.6 ± 53.2 G/L. The mean absolute eosinophil count was 2.0 ± 0.6 G/L. Based on these values, we calculated the mean NLR and PLR as 2.2 ± 0.9 and 142.2 ± 49.3, respectively. Moreover, the median values were 1.9 and 131.2, respectively, and were used for the stratification of the group into high and low. The high group (> median) contained 32 patients (49.2%), and 33 patients had a low (≤ median) NLR or PLR value (50.8%).

### Evaluation of the prognostic value of NLR and PLR with regards to NPS

Prior to binary logistic regression, we conducted a Chi-Square test in order to gain insights into the prognostic values of NLR and PLR. In particular, we tested if NLR and PLR were associated with therapy response in terms of total NPS improvement. Indeed, high PLR was associated with a higher rate of total response (62.5%, n = 20/32 vs. 36.4%, n = 12/33; p = 0.035). On the other hand, high NLR was not associated with a higher rate of total therapy response (56.3%, n = 18/32 vs. 42.4%, n = 14/33; p = 0.265).

### Prognostic relevance of PLR in terms of subjective outcome measures

Next, we assessed the prognostic relevance of PLR with regards to SNOT-22 and VAS smell function and nasal breathing and observed no significant associations of the pretreatment PLR with subjective measures. In particular, the contingency tables showed no significant differences in distribution of high and low improvements in the SNOT-22 among groups of high and low pretreatment PLR (p = 0.397) as well as in VAS smell function and nasal breathing (p = 0.293 and p = 0.144, respectively). Similar, no prognostic relevance of PLR was revealed for smell function improvement as shown by Sniffin’ sticks (p = 0.642).

### Univariable and multivariable analyses

In order to independently test the prognostic capacity of NLR and PLR in terms of predicting the total therapy response to dupilumab, we conducted an analysis with binary logistic regression. Only high PLR significantly correlated with the rate of total NPS reduction. Therefore, we included PLR in a multivariable model adjusted for age, sex, the pretreatment NPS score, asthma, AERD, total platelet and neutrophils count, and the number of previous ESS, and presented these results in Table 3. Furthermore, as the eosinophilia potentially reflects the type 2 inflammation, the total eosinophil count was also included in the binary logistic regression. Indeed, the high pretreatment PLR was an independent risk factor for complete NPS improvement (Multivariable analysis for high PLR: odds ratio 3.9 for achieving the NPS 0, 95% confidence interval (CI) 1.1–14.7, p = 0.04). As noted, no statistically significant results with regard to pretreatment NLR were observed (Univariable analysis for high NLR: odds ratio 1.7 for achieving the NPS 0, 95% CI 1.7–4.7, p = 0.27). In conclusion, patients with a pretreatment PLR > 131.2 had an almost fourfold higher chance of reaching the NPS of 0 after at least eight weeks after the start of dupilumab treatment.

**Table 3:** Binary logistic regression showing the prognostic value of inflammation markers

Risk factors for total NPS improvement	Univariable analysis			Multivariable analysis		
	Odds ratio	95% CI	p value	Odds ratio	95% CI	p value
Pretreatment PLR (high vs. low)	2.9	1.1–8.0	0.04	3.9	1.1–14.7	0.04
Pretreatment NLR (high vs. low)	1.7	0.7–4.7	0.27			
Eosinophils (metric)	0.5	0.1–6.3	0.61			
Neutrophils (metric)	0.9	0.7–1.4	0.75			
Plateletets (metric)	1.0	1.0–1.0	0.73			
AERD (yes vs. no)	2.4	0.9–6.7	0.08	1.8	0.5–7.3	0.40
Asthma (yes vs. no)	2.3	0.8–6.7	0.13	1.6	0.4–6.8	0.49
ESS (metric)	1.1	0.8–1.6	0.59	1.0	0.7–1.6	0.90
Age (metric)	1.0	1.0–1.1	0.51	1.0	0.9–1.0	0.24
Sex (male vs. female)	1.4	0.5–4.0	0.49	1.9	0.5–7.9	0.37
Pretreatment NPS (metric)	0.8	0.6–1.0	0.07	0.8	0.6–1.1	0.29

Total eosinophil, platelet, and neutrophil counts are presented in Giga/Liter

*NPS* nasal polyp score, *NLR* neutrophil-to-lymphocyte ratio, *PLR* platelet-to-lymphocyte ratio, *CI* confidence interval, *AERD* aspirin-exacerbated respiratory disease, *ESS* number of previous endoscopic sinus surgeries

## Discussion

Although showing excellent outcomes and treatment responses in patients with CRSwNP [15], a subset of patients does not respond to treatment with dupilumab, as assessed with objective as well as patient-reported outcomes [12]. This emphasizes the need for predictive biomarkers of therapy response of dupilumab in CRSwNP. In the current study, we were able to observe an almost fourfold increased probability of reaching the post-treatment NPS value of 0 in patients with a high pretreatment PLR (> 131.2). The prognostic relevance of pretreatment NLR was not statistically significant. Importantly, confounders possibly indicating a non-type 2 respiratory disease were included in the analysis and showed no significant impact on the outcome.

The association of high pretreatment NLR and PLR values with poor outcome has already been presented for different malignant diseases, mainly explained as the reflection of the enhanced tumor-related systemic inflammation [16–18]. Even in sinonasal cancer, high NLR and PLR were reported in patients with worse survival outcomes in the study of Turri-Zanoni et al. [19]. However, besides two authors assessing the values of NLR and PLR in rhinosinusitis patients, no studies could be identified with regard to the prognostic value of these markers in CRSwNP patients treated with dupilumab. In contrast to results in cancer patients, high value of PLR seems to correlate with better therapy response to dupilumab in CRSwNP. Interestingly, high NLR was associated with a higher recurrence rate after ESS in CRSwNP, and the PLR was higher in patients with recurrence [10].

As noted, these ratios potentially reflect the degree of systemic inflammation. Several authors aimed to explain the underlining mechanism between inflammation and the values of these parameters [10, 20, 21]. Generally, systemic inflammation is mostly marked by a high number of neutrophils [10]. Furthermore, different inflammation mediators lead to an increased absolute platelet count [20]. Regarding the second component of both ratios, lymphocytes are involved in the regulation of different inflammatory processes. As furthermore noted, a low number of lymphocytes is usually observed simultaneously with systemic neutrophilia [21]. Therefore, high NLR and PLR should be associated with a higher degree of systemic inflammatory processes. However, the exact correlation with the type 2 inflammation in CRSwNP with these markers remains unclear and warrants further elucidation.

Therefore, the observed correlation of high PLR with total therapy response to dupilumab marks a novel finding. As noted, higher PLR was observed in patients with CRSwNP recurrence after ESS [15]. On the contrary, high PLR prior to dupilumab therapy was associated with a higher rate of the total response to dupilumab in our study (defined as post-treatment NPS of 0). Based on these findings, it could be hypothesized that a higher rate of systemic inflammation caused by the CRSwNP, as reflected by high PLR, warrants a systemic treatment rather than surgical therapy. Indeed, the underlying mechanistic effects should be further elucidated. It remains unsettled, why high PLR predicts better response to dupilumab, particularly due to the fact that high PLR mostly associated with worse outcome. Moreover, our findings certainly need to be validated in order to make a definitive statement. Importantly, we measured therapy response only by the polyp size reduction. Furthermore, the prognostic value of inflammatory markers including PLR should certainly be investigated in other conditions treated with dupilumab, such as asthma or atopic dermatitis.

The proper assessment of the therapy response to biologics, including dupilumab, has been a subject of discussion. The European Forum for Research and Education in Allergy and Airway Diseases has published a consensus paper that includes a proposed assessment of response to therapy with biologics [22]. In particular, they noted that the following factors reflected the treatment response: reduction of polyp size, reduced need for corticosteroids, improvement in olfactory perception, improvement quality of life, and reduction of the impact of comorbidities. Interestingly, they proposed the first evaluation after 16 weeks, followed by a definitive evaluation after one year. Similarly, one group assessed the therapy outcome post dupilumab treatment after 16 weeks [23]. On the other hand, Hopkins et al. [24] assessed subjective and objective outcomes 24 weeks after the dupilumab start in CRSwNP. However, the biggest NPS improvement was noticed during the first 8 weeks of dupilumab treatment in the SINUS-24 study [11]. As the primary endpoint of our study was the improvement of the NPS, we therefore included the 8-week time-point as post-therapeutic. Furthermore, the follow-up data afterwards was limited in our cohort.

The conclusions of the current study can be challenged by different limitations. First, the timepoint defined as post-treatment was not the same in all patients. Thus, the impact of different time points and duration of treatment on the response warrants further investigation, particularly the outcome 16 weeks after treatment start. Due to the retrospective nature of the study, a potential selection bias could not be excluded. Notably, we excluded the effects of several confounders in the multivariable analysis; however, other confounders could have an effect as well. Importantly, the mechanistic underlying effects behind our observation remain unclear and warrant further elucidation. Moreover, according to the clinical markers and PROMs, the majority of our patients had a milder CRSwNP, and would have possibly not be prescribed a biologic in other areas of the world. Last, the size of the patient cohort is limited, potentially contributing to the missing of some significant effects and to wide CI values. In particular, significant association between PLR and subjective outcomes could have been missed, especially due to missing values in several patients. However, as this is a novel treatment, the majority of studies on this subject included small cohorts. Indeed, large-scale, multicentric investigations are certainly needed in order to make a definitive statement.

## Conclusion

Based on the findings of the current study, it seems that PLR might have a predictive value in CRSwNP patients in terms of treatment response to dupilumab. In particular, PLR above 131.2 was significantly associated with the complete elimination of nasal polyps after at least eight weeks of treatment. In order to make a definitive statement, the calculated cut-off value needs external validation, particularly in cohorts with severe CRSwNP. Furthermore, the exact prognostic value of PLR for patients with CRSwNP warrants further investigation in a large-scale cohort, examining further objective and subjective outcomes measures.

## Data Availability

All data generated or analysed during this study are included in this published article.

## Abbreviations

CRSwNP: Chronic rhinosinusitis with nasal polyps
Covid19: Coronavirus disease
ESS: Endoscopic sinus surgery
NLR: Neutrophil-to-lymphocyte ratio
PLR: Platelet-to-lymphocyte ratio
MLR: Monocyte-to-lymphocyte ratio
USA: United States of America
NPS: Nasal polyp score
G: Giga
L: Liter
AERD: Aspirin-exacerbated respiratory disease
